# Examining Cognitive Function and Sleep Disturbances in Adults With Schizophrenia Using Mobile and Wearable Sensors

**DOI:** 10.1093/geroni/igaf122.312

**Published:** 2025-12-31

**Authors:** Ellen Lee, Jerry McDonald, Andrea Coppola, Stephanie Ibrahim

**Affiliations:** University of California, San Diego, San Diego, California, United States; University of California, San Diego, San Diego, California, United States; VA San Diego Health Care System, San Diego, California, United States; University of California, San Diego, San Diego, California, United States

## Abstract

Due to cognitive impairment during the prodromal period/onset of schizophrenia and accelerated brain aging, older people living with schizophrenia (PLWS) have 10- to 20-fold higher prevalence of dementia, compared to the general population. While risk factors for dementia (e.g., metabolic side effects of medications and sedentary behaviors) can be difficult to treat, addressing sleep disturbances could improve or preserve cognitive functioning among PLWS. Our group has been assessing outpatient middle-aged and older PLWS with wearable sleep trackers, home sleep tests, and mobile cognitive testing to understand the complex relationships of sleep and cognition. We will present findings comparing mobile cognitive testing tasks with traditional neuropsychological battery (MATRICS). We will present associations of cognitive functioning (mobile cognitive testing and MATRICS battery) with sleep measures among PLWS and age- and sex-comparable non-psychiatric comparison (NC) participants. We will explore the relationships of sleep with mental and physical health measures. Our initial analyses (subsample of 12 PLWS and 28 NCs) found strong correlations between later mean wake-time and better scores on attention, working memory, and processing speed tasks (rs=.62-.78, p’s<.05, n = 12), between longer total sleep time and less variable scores on working memory task (rs=.72, p=.008, n = 12) only in PLWS. We did not observe these relationships among the NC participants. Study recruitment and assessment is ongoing, and we will present the updated results on a larger sample at the conference.

